# Growth hormone responsiveness: why does it vary and can it be predicted?

**DOI:** 10.1186/1687-9856-2015-S1-O5

**Published:** 2015-04-28

**Authors:** Wayne S Cutfield

**Affiliations:** 1 Liggins Institute, University of Auckland, Auckland, New Zealand

## 

Initial response to growth hormone (GH) varies considerably. In severe GH deficiency initial response varies across the group by almost 100%. In common non-GH deficient disorders such as idiopathic short stature (ISS) and children born small for gestational age (SGA) initial response varies by more than 100%.

The explanation for the wide response to GH treatment can be clustered into three broad groups; (i) non-compliance with GH therapy, (ii) clinical characteristics associated with GH responsiveness and (iii) mutations and common gene variants in the GH-IGF-I axis.

(i) Non-compliance with GH therapy: The commonest explanation for variable GH response is missed GH doses. In a national prospective study of GH compliance in New Zealand children; 34% of children missed more than one GH dose per week. Maori and Pacific children missed an additional GH dose each week than Caucasian children. Predictably, poor compliance (≥4 doses/week missed) was associated with a far poorer growth response than good compliance (height velocity SDS +0.4 vs +2.8). Even moderate compliance (2-3 doses/week missed) was associated with a poorer growth response (height velocity SDS +1.4 versus +2.8). Interestingly, paediatricians were unaware of GH non-compliance. GH response can only be maximised when more effective GH delivery devices are developed that record the date and time GH is administered to enable paediatricians to more effectively address compliance.

(ii) Clinical characteristics associated with GH responsiveness: There is a differential response to GH therapy according to the underlying disorder. GH deficient children are more responsive than those with Turner Syndrome or idiopathic short stature who are in turn more responsive than SGA children (as shown by height velocity and serum IGF-I for a given GH dose). Furthermore, across the normal birth weight range we have recently shown that progressive reduction in birth weight SDS is associated with a progressively lower IGF-I response during IGF-I generation testing. Across growth disorders; more severe GH deficiency, taller parents, shorter stature, younger age at the start of treatment and heavier children are all parameters found to be associated with greater initial and sustained response to GH treatment. Michael Ranke has pioneered individualised GH dosing through GH prediction modelling across growth disorders. GH response can be predicted by inserting common clinical characteristics into a prediction equation that includes adjusting the dose. Prediction modelling is of greatest benefit to children with adverse clinical characteristics in whom greater initial GH doses will lead to greater growth response.

(iii) Mutations and common gene variants in the GH-IGF-I axis: Downstream gene mutations in this axis (GH receptor, IGF-I and IGF-I receptor) are relatively rare causes of short stature, poor growth and GH response. Conversely common gene variants are being increasingly sought that influence stature, growth and GH responsiveness. Initial reports suggested a common GH receptor exon 3 deletion was associated with a better response to GH than the full length form in SGA and ISS children. Further studies have found little if any effect of the GH receptor deletion on initial or sustained growth response in SGA, Turner Syndrome or GH deficiency. Future studies are likely to focus on panels of common gene variants across the GH axis rather than examining individual genes to better characterise GH responsiveness.

